# From Coercion to Physical Force: Aggressive Strategies Used by Women Against Men in “Forced-to-Penetrate” Cases in the UK

**DOI:** 10.1007/s10508-018-1232-5

**Published:** 2018-08-02

**Authors:** Siobhan Weare

**Affiliations:** The Law School, Bowland North, Lancaster University, Lancaster, LA1 4YN UK

**Keywords:** Forced-to-penetrate, Sexual violence, Male victims, Female perpetrators, Gender

## Abstract

“Forced-to-penetrate” cases involve a man being forced-to-penetrate, with his penis and without his consent, a woman’s vagina, anus, or mouth. This article presents the first quantitative and qualitative research findings regarding such cases in the UK, exploring aggressive strategies used by women, as reported by 154 men who experienced them. The most frequently used strategies include coercion, taking advantage of men’s intoxication, and the use of force and threats of physical harm. Novel evidence is presented of women combining multiple strategies within the same incident. The article also argues that some of the strategies used by women are particularly “gendered,” with them taking advantage of their roles as women. The findings presented here raise questions for criminal justice professionals working in the area of sexual violence, as well as highlighting the need for future research.

## Introduction

Sexual aggression encompasses a broad range of tactics “designed to result in sexual interaction with an individual against [their] will” (Oswald & Holmgreen, 2013, p. 77). It includes verbally and physically coercive behavior, the exploitation of an individual under the influence of drugs or alcohol, and the active use of alcohol or drugs to engage in non-consensual sexual activity (Oswald & Holmgreen, 2013). Existing research highlights that sexual aggression and violence are overwhelmingly and disproportionately perpetrated by men against women, resulting in, understandably and quite correctly, a large body of research considering male sexual aggression. However, female sexual aggression, specifically toward adult men, and particularly more serious forms of such aggression (e.g., forced intercourse), have been excluded almost entirely from the research agenda. No studies of women’s sexually aggressive strategies in relation to compelled penetration have been conducted in the UK, and therefore, there is no indication of the prevalence of this form of sexual violence, nor the experiences of male victims. This is despite evidence from other jurisdictions of forced intercourse being an issue (see, e.g., Krahé, Waizenhöfer, & Möller, 2003; Struckman-Johnson & Struckman-Johnson, 1998; Tomaszewska & Krahé, 2018).

This article makes a novel and significant contribution to knowledge by sharing quantitative and qualitative data collected from the first study in the UK exploring experiences of men who have been forced-to-penetrate (FTP) women. These cases involve a man being FTP a woman’s vagina, anus, or mouth using his penis and without his consent (Weare, 2018). The existing legal definitions of rape within the UK exclude FTP cases. Indeed, within England and Wales^1^ for example, in s1 of the Sexual Offences Act 2003, rape is defined as the non-consensual and intentional penile penetration of the vagina, anus, or mouth of the victim without a reasonable belief in consent. The requirement of penile penetration of the victim excludes FTP cases from prosecution as rape. Instead, FTP cases can be prosecuted under the offenses of “sexual assault” (The Sexual Offences Act 2003, s3) or “causing a person to engage in sexual activity without consent” (The Sexual Offences Act 2003, s4), both of which are “less serious” offenses, typically attracting shorter sentences, within the legal framework.

By exploring women’s sexual aggression in the UK in relation to a previously unexplored form of sexual violence (compelled penetration), this article goes someway to filling a significant gap in the existing literature and helping to develop understandings of FTP cases as a specific form of sexual violence. As well as reporting on the frequencies with which aggressive strategies are used by women in FTP cases, evidence is presented that demonstrates how some women combine multiple aggressive strategies in the same incident. It is also argued that some women use especially “gendered” strategies that take advantage of their roles as women, namely threats of false rape allegations and interference in the father–child relationship in their roles as mothers.

Before proceeding, it is worth noting that despite ongoing assumptions that a man physically cannot be FTP a woman without his consent, it is in fact biologically possible for this to occur. Indeed, research has consistently reported that men can obtain and sustain erections even where they are not sexually aroused and/or are experiencing negative emotions such as anxiety, fear, or terror (see, e.g., Sarrel & Masters, 1982). Indeed, it is evident that “an erection can be induced by fear and is not necessarily indicative of pleasure or consent. Such heightened emotions can created unwanted arousal in men and if stimulated, in some cases, ejaculation can occur” (Fisher & Pina, 2013, p. 57). Therefore, sexually responding to a woman’s advances or touch does not automatically denote arousal or consent on the part of a man.

The terminology used within this article should also be noted, with the terms “sexual aggression” and “aggressive strategies” being purposely used, despite arguably being controversial, and, for some, problematic. This is for two reasons: firstly, within England and Wales the offense of Coercive Control has been introduced into law by s76 Serious Crime Act 2015, meaning that coercion now has a more specific legal meaning in the main jurisdiction to which the findings presented here are relevant. Secondly, coercion is one of the specific strategies used by women as identified by men in their narratives. Therefore, in this article “sexual aggression” is used to denote sexual conduct which is unwanted and performed against a person’s will, but that is not limited to the use of force. “Aggression” denotes the non-consensual nature of the experience.

While the focus of this article is on aggressive strategies used by women in FTP cases, claims are not being made as to the potential prevalence of this form of sexual violence within the UK. This is because no data currently exist within the UK on the prevalence of FTP cases, and the use of a purposive, rather than random or stratified sample in this study, means that prevalence rates cannot be assessed. However, it is noteworthy that recent prevalence rates on men’s experiences of sexual aggression from women (including compelled penetration) have been noted elsewhere. For example, Tomaszewska and Krahé’s (2018) study on sexual victimization and perpetration among Polish university students found a self-reported victimization rate of 28.4% for men (Tomaszewska & Krahé, 2018). Similarly, Krahé and Berger’s (2013) study on sexual perpetration and victimization among university students in Germany reported that 17.1% of men self-reported victimization from a woman (Krahé & Berger, 2013). Looking specifically at the prevalence of compelled penetration, in the U.S., data from the large-scale *National Intimate Partner and Sexual Violence Survey 2010* found a lifetime prevalence rate for men of 4.8%, with 79.2% involving a female perpetrator (Black et al., 2011, pp. 2 and 24).

Finally, it should be noted that the discussion and analysis of women’s aggressive strategies in this article are not undertaken comparatively to those reported as being used by male perpetrators in sexual violence and rape cases. This is because the article is not aiming to ascertain gender (a)symmetry in relation to strategies used by perpetrators or experienced by victims. Rather the aim is to explore FTP cases as a specific type of sexual violence, exploring female perpetrators’ aggressive strategies as experienced by male victims. Consideration is also not given to the consequences for men of the aggressive strategies used by women, e.g., physical or emotional harms. While such data have been collected in this study, exploration of it will be a focus of future work.

### Literature Review

#### Existing Research on Female Perpetrated Sexual Violence Against Adult Men

Although research studies have focused on aggressive strategies used by women perpetrating sexual violence against men, none of the existing research has looked specifically at FTP cases. These cases have often been excluded from consideration altogether (see, e.g., Anderson & Aymami, 1993), but where they have been considered it has either been alongside an exploration of a range of other non-consensual sexual acts perpetrated by women (see, e.g., Struckman-Johnson & Struckman-Johnson, 1998), in the context of a comparative approach also looking at forced sex experiences of women (Struckman-Johnson, 1988), or in some cases, combinations of the above (see, e.g., Tomaszewska & Krahé, 2018). As such, it is almost impossible to paint any sort of accurate picture around sexually aggressive strategies used by women specifically in FTP cases.

Studies which do consider FTP cases are, unfortunately, limited in number and scope. Most studies have been conducted in the U.S. and are largely outdated, with the majority from the 1980s and 1990s (see, e.g., Muehlenhard & Cook, 1988; O’Sullivan, Byers, & Finkelman, 1998). A few, more recent studies have been conducted in Europe, but, as noted above, they explore the issue of compelled penetration alongside other forms of sexual violence experienced and perpetrated by both men and women (see, e.g., Krahé & Berger, 2013; Krahé et al., 2015; Tomaszewska & Krahé, 2018). Further limitations with the existing research include the use of different definitions of sexual aggression and violence (Fisher & Pina, 2013), methodological inconsistencies (Byers & O’Sullivan, 1998), and the examination of specific male populations, typically college/university students (Davies & Rogers, 2006). It is also notable that existing research in this area is overwhelmingly quantitative in nature, thus limiting the more detailed understanding that is often revealed only when qualitative data are also included.

Despite the dearth of research in this area and the limitations associated with existing studies, it is possible to identify some general trends in relation to the aggressive strategies used by women by exploring the broader sexual violence literature (see, e.g., Bouffard, Bouffard, & Miller, 2016). The majority of these studies have looked at other forms of sexual violence perpetrated by women toward men, not just compelled penetration. These trends are based upon studies reporting the experiences of men, as well as self-reporting by women who have used aggressive strategies. Among the most frequently reported strategy experienced by men is verbal pressure, persuasion, or coercion (Muehlenhard & Cook, 1988; Struckman-Johnson, Struckman-Johnson, & Anderson, 2003). Women have also self-reported using coercive strategies with high frequency (Anderson, 1998; Struckman-Johnson et al., 2003). Alcohol and/or drugs also feature highly in the aggressive strategies reported by male victims and by female aggressors (Anderson, 1998; Struckman-Johnson et al., 2003). This most often takes the form of women exploiting already intoxicated men to engage them in sexual activity (see, e.g., Tomaszewska & Krahé, 2018), but there is also evidence of women actively intoxicating men to carry out sexual activity (see, e.g., Anderson & Aymami, 1993). Regardless of the form taken, “the role of alcohol in the context of sexual aggression is clearly demonstrated” (Krahé & Berger, 2013) in existing studies. In contrast, the use of physical force or weapons (including threats of) has most frequently featured as among the least common strategy in both reports by men and self-reporting by women (Struckman-Johnson et al., 2003).

In summary, although some general trends in the aggressive strategies of women perpetrating sexual violence against men have emerged, the limitations of the research conducted in this area mean that knowledge and understanding are limited, particularly regarding FTP cases. The shortcomings of the existing research have been acknowledged by several academics, and numerous calls have been made for more studies in this area to be conducted (Fisher & Pina, 2013; Stemple, Flores, & Meyer, 2016; Weare, 2018). As such, this study and the findings presented here begin to address several current gaps in knowledge and understanding relating to: (1) the experiences of men FTP women in the UK and (2) the aggressive strategies used by women in such cases in the UK. It is notable that the inclusion of both qualitative and quantitative data to explore the issue of compelled penetration is also novel, thus addressing a further gap within the existing literature, where a more detailed understanding of this form of sexual violence is required. The mixed-methods approach taken in this study allows for critique of existing debates around the use of specific aggressive strategies by women, as well as an exploration of previously unconsidered issues raised within participants’ narratives.

## Method

### Participants, Procedure, and Measures

This study investigated the experiences of men, through their own narratives, who had been FTP women. Data were collected for 8 weeks between December 2016 and the end of January 2017 using an online survey, hosted by *SurveyMonkey.* This was chosen as the most appropriate method of data collection for two key reasons: to preserve participant anonymity and to maximize the number of participants. Using an online survey was the easiest way to preserve anonymity as participants did not need to meet with the researcher or disclose identifying information. While anonymity meant that participant identities and the veracity of answers could not be confirmed, the sensitive nature of the issues under consideration meant that the ability to disclose experiences anonymously increased not only the likelihood of men participating, but also them providing truthful and detailed accounts of their experiences that lacked embellishment. Indeed, the “hidden–hidden” nature of FTP cases and the complex gender dynamics involved were important methodological considerations. It is also noteworthy that anonymity in relation to sexual violence is reflected in law in the UK, with lifetime anonymity being guaranteed to victims of rape or serious sexual assault under s1 of the Sexual Offences (Amendment) Act 1992. Anonymity for participants in the study extended not only to participation, but also to withdrawal. Participants were able to provide non-identifying “safe words” which could be emailed to the researcher should they wish to anonymously withdraw from the survey. Instructions on setting up temporary email addresses were provided as a further safeguard to preserve anonymity.

The use of an online survey also maximized participation. Hosting the survey online allowed participants to be recruited via media publicity, both online and in print form, from across the UK (e.g., in local and national newspapers and on news websites). The survey was shared widely on social media (e.g., Twitter). Details of the survey were also distributed via email to organizations working with men who had experienced sexual violence (e.g., Survivors Manchester and ManKind Initiative), who then forwarded the details on to potential participants as appropriate. A project website was set up to provide information to potential participants. Prior to completion of the survey, participants were provided with an information page containing detailed information about the scope of the survey. In advertising and describing the survey, the term “forced-to-penetrate” was defined as encompassing any, and all, cases where a man engages in penile penetration of a woman without his consent and could include non-consensual penile penetration of a woman’s vagina, mouth, or anus. To try and prevent response (and non-response) biases, several different examples of such circumstances were provided, with it being made clear that these formed only part of a non-exhaustive list. Potential participant numbers were unknown due to the absence of previous research on this issue within the UK, and so an online survey provided the flexibility needed to engage with this unknown factor.

A mixed-methods approach to data analysis was taken with the collection of both quantitative and qualitative data in relation to the aggressive strategies used by female perpetrators. Participants were presented with a series of options relating to strategies (see Table 6) and were asked to select the one which most closely reflected that used by the woman in their most recent experience. The list of options was adapted from the *Sexual Experiences Survey*—*Short Forms Victimization* (SES-SFV) (Koss et al., 2006). Participants were then presented with an open-ended follow-up question asking them to describe, in as much detail as possible, what happened during their most recent experience.

The quantitative data underwent frequency and descriptive analysis to determine which aggressive strategies were most often reported by participants (see Table 6). Cross-tabulations were used to determine whether there were significant relationships between participant demographics (e.g., age at the time of their most recent FTP experience, relationship with the perpetrator) and the aggressive strategies reported. No significant relationships were found between any of these variables.

The qualitative data gathered from the open-ended follow-up question were open-coded using NVivo.^2^ Content analysis was undertaken to allow sub-categorizations to be developed in relation to each of the individual aggressive strategy options. Once the data had been organized in this way, thematic analysis was used to identify key patterns and thematic structures in respect of the aggressive strategies and to draw out emerging narratives as detailed by participants. The themes identified under each strategy were then compared to ascertain whether any overlaps appeared in the thematic structures identified. Where overlaps were identified, this has been reflected in the merging together of relevant strategies in the research findings section below.

The study was reviewed and approved by the research ethics board at the author’s university. Guidance and support were offered to participants with contact information for specialist support organizations provided before and after completing the survey. The questions in the survey were qualitative and quantitative in nature and consisted of closed questions (e.g., discrete options or a range of choices) and open-ended ones, focusing principally on the men’s most recent FTP experiences. Focusing on most recent experiences, rather than, for example, “worst” experiences, was a conscious decision in an attempt to represent a “typical” experience of sexual aggression from a woman (O’Sullivan et al., 1998). Participants could skip questions they did not want to answer to minimize distress from recounting possibly traumatic and intensely personal experiences. The survey asked the same substantive questions of all participants; however, they could take slightly different “routes” through the survey, depending on both the questions answered and the answers given to certain questions. These two factors meant answers to every question were not provided by every participant. Consequently, details are provided regarding the number of responses given to the questions discussed below. Participants were asked a range of questions in relation to their most recent FTP experience; however, this article is only going to focus on those relating to the aggressive strategies used by women.

Participants were self-selected, identifying themselves as having been FTP a woman. As a result, the sample is not representative of all men who have experienced compelled penetration, nor their experiences. It is, however, as representative a group as might conceivably be collected when considering the research methods used, the sensitive nature of the issues under consideration, and the “hidden–hidden” nature of this form of sexual violence. This should also be considered in the context of the research aims which were to explore and understand the experiences of men, including the aggressive strategies used by women, in FTP cases, and to do so through men’s own narratives. A total of 159 men participated in the study, resulting in a usable dataset involving 154 participants. Five of the surveys were removed because they were clearly completed as hoaxes based on the “infantile,” and sometimes incoherent, answers provided. For example, in response to the question asking participants to describe what happened during their most recent FTP experience, one response was, “Very Sexy Women.”

Participants were asked to provide demographic information on their age at the time of completing the survey, their age at the time of their most recent FTP experience, their sexual orientation, and country and region of residence. A total of 148 men provided their age at the time of completing the survey, with the average being 38 years. The youngest participant was 18 years old (the minimum age to participate in the survey), and the oldest was 70 (see Table 1). Based on 153 responses, the average age of participants during their most recent FTP experience was 27 years, with a range of between two and 61 years (see Table 2). In terms of sexuality, 134 of the 154 (87%) men who answered this question described themselves as heterosexual, 17 (11%) as bisexual/bicurious, and three (2%) as homosexual. All 154 participants disclosed their country of residence, coming from across all four countries within the UK, but with the highest number from England (72.1%) (see Table 3). Of the 129 who disclosed an area or region of residence, the South East featured most frequently (28.7%). This is perhaps unsurprising as the area of the UK with the highest population density. The only areas in the UK not identified as areas of residence were East Wales and North Scotland (see Table 4).

**Table 1:** Age at the time of completing survey—grouped participant age ranges (frequency)

Current age (years)	Frequency	Percentage
18–25	24	16.2
26–35	48	32.4
36–45	39	26.3
46–55	25	16.9
56–65	10	6.8
66 +	2	1.4
Total	148	100

From S. Weare, Forced-to-penetrate cases: Lived experiences of men. Copyright 2017 by Siobhan Weare

**Table 2:** Age during most recent FTP experience—grouped participant age ranges (frequency)

Age during most recent FTP experience (years)	Frequency	Percentage
≤ 15	16	10.5
16–25	67	43.8
26–35	36	23.5
36–45	21	13.7
46–55	11	7.2
56 +	2	1.3
Total	153	100

From S. Weare, Forced-to-penetrate cases: Lived experiences of men. Copyright 2017 by Siobhan Weare

**Table 3:** Country of residence (frequency)

Country of residence in the UK	Frequency	Percentage
England	111	72.1
Wales	7	4.5
Scotland	30	19.5
Northern Ireland	6	3.9
Total	154	100

From S. Weare, Forced-to-penetrate cases: Lived experiences of men. Copyright 2017 by Siobhan Weare

**Table 4:** Region of residence (frequency)

Region of residence in the UK	Frequency	Percentage
North West England	20	15.5
North East England	10	7.8
South East England	37	28.7
South West England	11	8.5
East Midlands England	7	5.4
West Midlands England	12	9.3
North Wales	3	2.3
South Wales	3	2.3
East Wales	0	0
West Wales	1	0.8
North Scotland	0	0
South Scotland	7	5.4
East Scotland	10	7.8
West Scotland	4	3.1
Northern Ireland	4	3.1
Total	129	100

From S. Weare, Forced-to-penetrate cases: Lived experiences of men. Copyright 2017 by Siobhan Weare

Participants were also asked about their relationship with the woman at the time of the compelled penetration incident they were reporting (see Table 5). All 154 participants answered this question with the majority disclosing that they knew the woman in some capacity. Seventy-nine men (51.3%) were in or had been in an intimate relationship (e.g., girlfriend/fiancée/wife) with the woman, and 43 (27.9%) indicated that they knew her as an acquaintance or friend.

**Table 5:** Relationship with female perpetrator during most recent FTP experience (frequency)

Relationship with female perpetrator	Frequency	Percentage
Wife/ex-wife	25	16.2
Ex-girlfriend/ex-fiancée	16	10.4
Girlfriend/fiancée	38	24.7
Relative	7	4.5
Acquaintance/friend	43	27.9
Stranger	7	4.5
Other	18	11.8
Total	154	100

From S. Weare, Forced-to-penetrate cases: Lived experiences of men. Copyright 2017 by Siobhan Weare

## Results

A total of 153 participants answered the quantitative question asking them to indicate which of the “options” presented (see Table 6) most closely matched their most recent FTP experience. The results are presented in Table 6 in order of descending frequency, rather than the order presented in the survey.

**Table 6:** Aggressive strategies used by female perpetrators (frequency)

Row	A woman forced you to penetrate her without your consent by:	Freq.	%
1	Telling lies, threatening to end the relationship, threatening to spread rumors about you, making promises you knew were untrue, or continually verbally pressuring you after you said you didn’t want to	34	22.2
2	Using force, for example, holding you down with their body weight, pinning your arms, restraining you, or having a weapon	22	14.4
3	None of the options present	22	14.4
4	Showing displeasure, criticizing your sexuality or attractiveness, getting angry but not using physical force, after you said you didn’t want to	17	11.1
5	Forcing you to penetrate her when you were asleep or unconscious from consensually drinking alcohol, and when you came to (regained consciousness) you could not give consent to or stop what was happening	17	11.1
6	Forcing you to penetrate her when you were asleep or unconscious from using drugs consensually and when you came to (regained consciousness) you could not stop what was happening	13	8.5
7	Forcing you to penetrate her after you had been drinking alcohol and were conscious but too intoxicated (drunk) to give your consent to or stop what was happening	11	7.2
8	Threatening to physically harm you or someone close to you	8	5.2
9	Acting together with two or more people to force you to penetrate her where you had made it clear that you did not give your consent to what was happening or were unable	4	2.6
10	Encouraging or pressuring you to drink alcohol until you were too intoxicated (drunk) to give consent to or stop what was happening	3	2
11	Giving you a drug without your knowledge that made you too incapacitated (out of it) to consent or stop what was happening	2	1.3
Total		153	100

From S. Weare, Forced-to-penetrate cases: Lived experiences of men. Copyright 2017 by Siobhan Weare

Ninety-seven men answered the open-ended follow-up question describing their experience. Varying amounts of detail were provided, from a couple of lines to several paragraphs. These quantitative and qualitative findings are discussed below. When presenting the research findings, the aggressive strategy “options” in Table 6 that are related or have overlap have been grouped together, for example the consensual consumption of alcohol and drugs (rows 5 and 6 in Table 6). It is recognized that the strategy “options” in Table 6 are discrete ones chosen by participants; however, the content and thematic analysis of the qualitative data produced some key patterns. Therefore, they are presented as grouped where appropriate to reflect this. The discrete “options” which have been grouped will be highlighted for clarity, and presented in order of descending frequency.

### Blackmail, Threats, Coercion, and Continual Verbal Pressure

This was the most frequent aggressive strategy used by women as reported by participants. Fifty-one (33.3%) of 153 men indicated this strategy was used across two discrete “options” in the survey—rows 1 and 4 in Table 6. Thirty-nine participants who selected the two “options” relating to coercive strategies provided additional details on their experiences. At its most extreme, this strategy involved women blackmailing and threatening men to force them into penetration. This took several forms. Two of the men were involved in consensual sexual affairs with the women outside of an existing relationship, but when they attempted to end them, the women threatened to tell friends and family about their infidelity unless they kept the sexual relationship going. For example:

After a few weeks I tried to end things as the relationship was toxic. She told me she didn’t want to end things as it was just sex and that if I did she would go to my wife and show her all our messages and describe to her in detail what went on. She then said she’d post pictures and more messages to all my work colleagues.

Another man reported that an ex-girlfriend threatened to lie to his current partner that they had had sex if he did not have intercourse with her:

I kept saying no, but she wouldn’t listen and threatened to tell my new girlfriend that we had had sex anyway and she threatened other things if I didn’t have sex with her. In the end she basically raped me.

Blackmail and threats also took other forms, with two participants reporting that they had been threatened with false rape allegations and two disclosing that suicide blackmail had been used by their current and former partners. For example:

Once inside [my apartment] she told me that if I didn’t have sex with her then she’d call the police and claim that I was trying to assault or rape her. She got her phone out and dialled 999 (but didn’t press “call”) to show me she was serious.

I had told the girl in question that I wished to end our relationship. She became extremely emotional and began saying that she was going to commit suicide because she couldn’t handle it. I have never seen someone so hysterical and distraught. I had had an ex-girlfriend try to commit suicide previously, and this girl knew that…

Another participant disclosed how a friend threatened self-harm and “to kill [her] unborn foetus if [he] didn’t comply.”

Threatening to stop a man seeing his children was also used by one woman. This was particularly coercive for the man who reported it as his ex-wife had previously stopped him seeing his children, and he had only just started seeing them again: “I didn’t want to have sex with her but feel I was made to by threats of not seeing my kids after so long. She was threatening to move her and the children…if she and I ‘couldn’t get along’.”

Threats to “out” particular sexual preferences occurred more than once, in particular, telling friends and family of sexual practices that could be viewed as deviant. For example, being on swinging websites or that the man cross-dressed (despite him reporting that this was for the woman’s sexual pleasure). One man also disclosed that his female employer threatened him with losing his job if he did not have sex with her.

Continual verbal pressure was also reported on several occasions and took multiple forms:

The psychological pressure she put me under, coupled with her constant communication by text, email and telephone, during which she told me that if I were a “proper man” I’d “prove it” and that I was “spiteful and selfish” for not giving her what she wanted, became so unbearable that I relented in the hope that “one last time” would indeed be that.

She wouldn’t take “no” for an answer. She wouldn’t stop. She wouldn’t leave me alone.

Emotional abuse and coercion were also reported by several of the men. For example:

I had to be ready for her whenever she wanted sexual intercourse, and was subjected to emotional abuse and blackmail if I didn’t give her what she wanted…[She] decided she wanted to have sex again, and used emotional blackmail and coercive tactics to make me comply, along the lines of telling me that I wasn’t satisfying her and I had to do it otherwise I wasn’t worth her time. I suffer from depression, and these tactics were very effective.

Finally, two participants reported that while no “active” coercion, in the form of spoken words, took place, there was a sense that there would be “consequences” for failure to comply; “I had no choice: if I did not penetrate her I would be punished for denying her,” “If I didn’t have sex I would be in ‘trouble’ and get silent treatment.”

### Taking Advantage of Men’s Intoxication via Drugs or Alcohol

Forty-one (26.8%) of 153 men reported this across three different strategy “options”—rows 5–7 in Table 6—making it the second most frequently experienced aggressive strategy. Twenty-five men who selected the three options grouped here provided further qualitative details about their experiences. Most frequently, participants reported that they were asleep or unconscious following alcohol or drug consumption and woke up to find themselves having non-consensual sex. Several participants only reported their own intoxication and did not suggest that the women were similarly under the influence of alcohol. For example:

My friend and I were clubbing and met two women. My friend got together with one of them but I didn’t fancy her friend and so wasn’t interested. Throughout the night she repeatedly kept trying to kiss me and touch me to which I kept saying no. We all ended up back at my flat…I was so out of it…my friend had to carry me into my bed which I don’t remember. I then woke up with her on top of me and I was inside her. I don’t remember much just flashes of memory of her bouncing on top of me.

In other men’s reports, it was clear that both they and the female aggressor were intoxicated when the incident took place. However, on all occasions it seemed as if the men were more intoxicated, so much so that they had fallen asleep or were unconscious due to their alcohol or drug consumption. For example:

We got drunk…I could barely see, but needed to sleep it off. When we got to hers, I collapsed on the bed, she started kissing me and putting her hands down my trousers, I gently said no and then passed out. I woke up naked, with her down on me, bleary eyed I gently pushed her away and said no…I fell asleep again, but curled up. I woke up again but this time she was on top of me holding my arms down and I realised I was inside her…I turned around and found 3 condoms (used) were on the floor next to me.

Other men reported being conscious but too intoxicated to consent to, or stop, what was happening. For example, one man noted how his friend took advantage of his intoxication:

Following a night drinking around town with a larger group of friends…I was certainly drunk. After leaving a club she carried me to a taxi and went back to her house, she put me on the bed and whilst she was undressing herself I vomited onto my shirt. She helped to take my shirt off, before unzipping my trousers and firstly giving me oral sex.

Another participant explained how his partner took advantage of him already being drunk to ply him with more alcohol so he was unable to reject her advances:

We were drinking. I got really drunk. She wanted to have sex. I said no but she persisted. She gave me more alcohol until I couldn’t talk properly anymore. Once I was at that point she took my pants off and played with it until I was erect. She then got on top of me and put it inside of her.

### Use of Force and Threats of Physical Harm

When combining the “options” of use of force and threats of physical harm (including to a close friend or relative) (rows 2 and 8 in Table 6), this is the third most frequently cited aggressive strategy, with 30/153 (19.6%) participants reporting this strategy. Seventeen participants elaborated on their experiences, highlighting how the use of force by women varied. The use of physical weight or strength as a means for women to overpower men featured most frequently. For example, “She used her size and weight to stop me leaving and then physically forced me to have intercourse with her” and:

I pretended to be asleep and hoped she would leave me alone. She got undressed and into bed then angrily got on top of me and held me down—she was the same height and weighed a lot more. I said no but she continued and was angry and drunk.

Another participant explained how his partner took advantage of his reduced physical strength following back surgery to use her physical strength to compel penetration.

Specific forms of force were also highlighted, for example, choking, as well as more general physical violence. Two men mentioned the use of weapons by women. For example, one participant who had experienced ongoing domestic and sexual violence from his partner noted how she used a variety of “household items as weapons, [including pinning him] to the stairs with a knife to [his] throat.”

Aside from use of force, threats of physical harm were also reported, typically toward the men themselves. These involved general threats of violence as well as threats to inflict specific types of violence. For example, “She referred to threats previously made in similar situations, these were both towards inflicting injuries/pain on myself from general “I will hurt you” threats to specific threats about causing injuries.” One man disclosed that threats were made by his partner toward his children; “[she] threatened to abandon [the] children or hurt them…when I refused sex.”

### None of the Options Present

Twenty-two (14.4%) of 153 participants indicated that their experiences did not match with any of the options listed (row 3 in Table 6) and of these, 15 provided further details about their experiences. Content analysis highlighted that some men could have categorized their experiences differently, selecting another of the more “specific” options available. However, in taking an approach of centralizing the men’s narratives, importance was attached to maintaining the categorization that they had given to their experiences. Thematic analysis of these narratives highlighted additional strategies that had not yet emerged. The first related to issues of power imbalance within familial relationships, as well as with family friends. This was the case for three men, who were all pubescent boys (Blanchard et al., 2009) when their most recent FTP experience occurred and who were FTP either a family member or family friend.

Mother came into bedroom, pulled back covers. I was on my back and she straddled me. Took my penis and after touching it to get an erection guided it into her.

My sister 4 years my elder made me…said it was normal and told me she would tell dad I tried to do it to her if I didn’t.

The woman was a family friend who was staying overnight in our home. She came into my bed and started, as I now know fondling and kissing me, she then had sex with me…She was my mum’s friend. I could never tell my mum.

There is no suggestion of physical force being used in any of these experiences, and minimal, if any, verbal coercion. However, it is suggested that a form of “indirect” coercion was experienced by these pubescent boys (Blanchard et al., 2009), who lacked power in the context of complex familial relationships to resist the women’s actions.

For two men, the aggressive strategy used by their ex-partners involved negatively interfering in the relationships that they had with their children. For example:

The mother of my son (ex-partner) would cause me difficulty with my relationship with my son if she was unhappy about my attitude or something I had done. In order for me to stop the arguments or fighting was for me to initiate letting her give me oral…I certainly didn’t always want it but felt it was the easiest way to stop fighting for the sake of my son.

These experiences differ slightly to one noted earlier where a man was threatened more specifically that he would not be able to see his children.

Two participants explained how they had fallen asleep and woken up to find a woman having sex with them. For one man this was his long-term partner; “Was at home in bed with my partner of 7 years…We went to bed as normal and about an hour later I awoke to find her naked on top of me ‘helping herself’ to vaginal sex.” For another it was a sexual partner; “I awoke to find someone with whom I had had consensual sex with the night before, had penetrated herself anally with my penis whilst I was asleep.” In both instances there was no mention of intoxication and thus these experiences can be distinguished from those discussed earlier and below where alcohol or drugs were involved.

Two more specific aggressive strategies were disclosed by participants. One man explained how his ex-partner “lied about being pregnant and said she’d get an abortion if [he] didn’t have sex with her.” Another described how he was engaging in a consensual act with his partner which then became non-consensual; “Engaged in consensual sexual activity and as I ejaculate she grabs my penis and forces it into her vaginally because she wants to ‘feel me cum’ even though I have explicitly said I don’t want to do so without a condom.”

### Actively Involved in Encouraging Intoxication via Alcohol or Drugs

This was one of the least frequently reported strategies, with 5/153 (3.3%) participants reporting this across two “options”—rows 10–11 in Table 6. Only two participants provided qualitative detail on their experiences, with both reporting that the female perpetrator was a teacher at their school. One of the men was given a drug by his teacher without his knowledge before being FTP her. He noted that this had happened at school, but did not provide further details. Another, who was 15 at the time, visited his teacher’s flat with friends and was encouraged to drink until he was too intoxicated to stop what was happening:

She offered cigs and alcoholic drinks and weed. She offered us a drink and I accepted it…She had been talking to us and encouraging us to drink more, I don’t know how much time had passed but the rest [of the kids] had gone and I was sitting on her sofa next to her. She kissed me and then instantly put my penis in her mouth, my jeans had already been undone which I didn’t remember doing or her doing. I was drunk and although I was not completely out of it, I was not able to react quick enough to do anything. She then came back up pushed me down on to the sofa and straddled herself on top of me and put my penis into her vagina.

### Multiple Perpetrators

This was the least frequently cited aggressive strategy used by women, accounting for the experience of 4/153 (2.6%) participants (row 9 in Table 6). None of the men who selected this option provided further detail on their experiences, and thus further analysis could not be undertaken.

## Discussion

### Developing Understanding and Challenging Stereotypes

The findings from this project “[a]dd to a body of research that is designed to ‘dismiss the myth of the nonaggressive woman on empirical grounds’” (Krahé et al., 2003, p. 228). By demonstrating empirically for the first time in the UK that this form of female perpetrated sexual violence occurs, the findings of this project directly contravene “the traditional belief that a woman cannot force a man to have sex” (Davies, 2013, pp. 93–94). This is important because despite some recognition within academic research of women’s ability to compel men into penetration, there is still a widespread societal belief, informed by gender stereotypes and the traditional sexual script, that:

specific [sex] roles are assigned to men and others are assigned to women. [This] excludes the image of women as sexual aggressors, initiating sex with men…and, at times, coercing their partners to engage in unwanted sexual activities…[as well as excluding] the image of men as sexually reluctant or as victims of sexual coercion (Byers & O’Sullivan, 1998, p. 146).

The widespread and pervasive nature of the gender schematized traditional sex script has been remarked upon by Davies (2002), who notes that “most people, including many psychologists, view the sexual assault of men by women as somewhat implausible.” Thus, this study, though not suggesting prevalence rates, provides empirical evidence of the existence of this issue for the first time within the UK, which in turn challenges gender stereotypes that suggest that this form of sexual violence cannot or does not happen.

The findings from this research support some of the existing findings on women’s sexually aggressive strategies toward men. It is difficult to directly compare the quantitative findings presented here with those from other studies, largely because of the different definitions used to refer to similar behavior. For example, the terms “verbal pressure” (Krahé & Berger, 2013), “persuasion” (Struckman-Johnson & Struckman-Johnson, 1994) and “psychological pressure” (Struckman-Johnson, 1988) are all seemingly used to refer to verbally coercive strategies. Moreover, differing methodological approaches have been taken across studies, making accurate comparisons challenging. Nevertheless, the frequency with which some of the aggressive strategies are used broadly reflects prevalence rates found in existing studies.

In relation to verbally coercive strategies, while there are differences in the reporting rates of men experiencing this strategy—varying between 20 and 70% across existing studies—this strategy consistently features as either the most or second most reported within the majority of studies (see, e.g., Struckman-Johnson & Struckman-Johnson, 1998; Struckman-Johnson et al., 2003). Similarly, although the rates for women’s self-reporting of this strategy are generally lower, at between 0.8 and 43%, coercive strategies still feature as among the most frequently used (see, e.g., Anderson, 1998). In this way, and to this extent, the finding that verbally coercive strategies were experienced most frequently by participants is broadly in line with existing research in this area. The exceptions to the above are the findings from Tomaszewska and Krahé’s (2018) study involving male and female university students in Poland and Krahé et al.’s (2015) study across 10 European countries (excluding the UK). In both studies “verbal pressure” was less frequently reported, being the least frequently reported strategy experienced by male victims in Tomaszewska and Krahé’s (2018) study, or the second least frequently reported in Krahé et al.’s (2015) study. An explanation for the divergence here is difficult to pinpoint, but again it may reflect the differences in methodological approaches, differences in participant demographics, or other variable factors. Acknowledging some divergence in findings where they arise is important when considering the potential for future research in the area.

In relation to alcohol, the findings presented here reflect its prominence within both the aggressive strategies used by female perpetrators and the experiences of victims of sexual violence (Krahé & Berger, 2013). Indeed, the quantitative findings from the present study on the strategy of taking advantage of an already intoxicated man broadly accord with those found in existing literature, in that this was typically the most, or second most frequently reported strategy, both by men (see, e.g., Struckman-Johnson et al., 2003; Tomaszewska & Krahé, 2018) and in self-reporting by women aggressors (see, e.g., Anderson, 1998). However, in relation to active alcohol or drug use (i.e., where the female perpetrator is actively involved in the intoxication of the male victim), within existing research, higher rates have been reported. Indeed, it has often featured as one of the more frequent strategies where cited (see, e.g., Anderson & Aymami, 1993; Struckman-Johnson & Struckman-Johnson, 1998). One explanation for the discrepancy could be the definitions and explanations used in the studies. Indeed, it is difficult to make comparisons where broad terms like “intoxication” (see, e.g., Struckman-Johnson, 1988) are used without the provision of wider context as to how the intoxication occurred. Moreover, most of the existing research has involved college students who are living in an environment where “alcohol and drug use are common parts of social activity” (O’Sullivan et al., 1998, p. 179) and thus this may explain the higher rates of its use within these studies.

While many of the findings from this study broadly align with existing research in the area, this research also presents challenges to existing understandings of FTP cases, as well as women’s sexual aggression toward men more broadly. This principally relates to women’s use of physical force or violence. The findings presented here in relation to the use of force contradict most earlier empirical studies, which suggest that women are unlikely to use physical force or violence as an aggressive strategy. Indeed, as noted above, most existing studies have put the rate at which physical force is used by women at between 2 and 10% (Weare, 2018) and it has typically featured as the least frequently used strategy. These findings can be contrasted with this study where 14.4% of the men reported the use of force and 19.6% reported use of force and threats of physical harm combined (see Table 6). There are, however, some exceptions, where reporting of this strategy has been at higher percentage rates that are closer to those seen in this study. For example, Struckman-Johnson et al. (2003) reported that 24.7% of the 275 college men in their study had experienced one or more forms of physical force in relation to sexual contact, and Anderson (1998) found that 20% of 461 college women self-reported using physical force to obtain sexual contact with a man. Nevertheless, in most existing studies, even those where reporting of physical force was above 20%, “[p]hysical force was the least commonly utilised tactic” (Bouffard et al., 2016, p. 2363). The exception to this being more recent European studies, where use or threats of physical force have featured as among the most frequent aggressive strategies reported by male victims of women’s sexual violence (see, e.g., Krahé et al., 2015; Tomaszewska & Krahé, 2018).

As noted above, the findings presented here, where physical force was the third most frequently reported strategy, contradict much of the existing research which suggests it is less common. There could be several explanations for the higher reporting rate of this strategy here, the first of which is that this study solely explored men’s experiences of compelled penetration. Therefore, the higher frequency with which force is used may be specific to this form of sexual violence. Similarly, as this is the first study to look at this issue in the UK, there may be cultural and social differences that impact upon women’s use of force. The design of the study could also have impacted the increased reporting of this aggressive strategy. The study was promoted as being about FTP cases, and the use of the term “force” here may have led to a response bias. That is to say that despite efforts to prevent such a response bias (as noted earlier in the article), men who were victims of a use of “force” by a female perpetrator may have been more likely to respond to the survey, than men who experienced other aggressive strategies (e.g., where their intoxication was taken advantage of). Finally, the participants in this study were self-selected, rather than a convenience sample (e.g., college students) as seen in most existing studies. Therefore, the demographics of those who participated could account for the higher reporting rate of this aggressive strategy. Regardless of the explanation offered, the findings highlight that more effort needs to be put into dispelling the stereotype that women cannot and do not use force when compelling men into penetration and, more broadly, the myth that women do not “have the size, strength, or ability to physically force a man to have sexual contact” (Struckman-Johnson & Anderson, 1998, p. 11). This is a damaging stereotype that is likely to negatively impact upon reporting rates and criminal justice and societal responses to this form of sexual violence.

It is clear both from these findings, and those presented elsewhere, that women use a variety of sexually aggressive strategies. However, the inclusion of qualitative data in this study has also allowed previously unobserved information to be uncovered about the strategies used by women in FTP cases. In particular, two original findings have emerged: first, some women use multiple aggressive strategies within the same incident and, secondly, some women use particularly “gendered” strategies. These findings will go some way to developing clearer understandings around women’s aggressive strategies when perpetrating sexual violence against adult men.

### Women’s Use of Multiple Aggressive Strategies

While quantitatively the men were asked to select the “option” that most closely matched their most recent FTP experience, thus suggesting the use of only one aggressive strategy per incident, content analysis of the answers provided to the open-ended follow-up question suggests otherwise. Indeed, the qualitative data highlights the use of multiple aggressive strategies by some women within the same incident. This is not something that has previously been noted within existing research on women’s sexual aggression, except in passing by Struckman-Johnson et al. (2003). The research findings from this study indicate that some women combine aggressive strategies when compelling penetration. For example, one participant described how his partner was both verbally and physically abusive:

My partner of the time came home from a night out with some female friends, she had been drinking and also taken cocaine. She demanded sex, I refused, she became initially verbally abusive and then went on to physically hit me landing a number of blows to the side of my head until I complied.

Two participants also explained how women took advantage of them as they slept and then used force or restraint to compel penetration. For example: “I woke from my sleep to find me handcuffed to the bed and her giving me oral sex, I told her to stop but she wouldn’t.” While it is interesting in and of itself to note the combinations of strategies used by women, the value of this discovery lies in revealing more detail than previously known about women’s aggressive strategies and thus the experiences of the men subject to it. This level of understanding is crucial to developing appropriate responses to such cases, which remain underreported and under-discussed.

### Women’s Use of “Gendered” Aggressive Strategies

The second key finding from this study relates to women’s use of what is termed “gendered” strategies; that is, strategies where women are aware of, and take advantage of, their gendered roles and experiences, *qua women*. In the findings, these strategies took two forms: threats regarding false rape allegations and exploitation of women’s roles as mothers to interfere in the father–child relationship.

#### Threats of False Rape Allegations

As noted earlier, two instances of women threatening to make false allegations of rape against men were reported, for example:

A threat of a false rape accusation…she kept on telling me that she would tell the police that I had raped her and ruin my family and my life.

It is important not to make generalizations about this specific strategy, not least because only two participants reported it as part of their experiences. Moreover, in discussing this as a particular strategy, it is in no way suggesting that the issue of false rape allegations (and threats of) should dominate, or in any way undermine, the issue of women as victims of rape and other forms of sexual violence. Rather what is being raised is the fact that this particular strategy has not previously been identified within research in this area, and therefore recognizing it as a potential issue for men who experience this in compelled penetration cases is important. Indeed, the similarities in the men’s stories suggest that this “gendered” strategy would benefit from further exploration to develop understandings around its use. It is also important to consider this strategy in relation to the impact that false reporting of rape (and threats of) has on the treatment of rape, and rape victims, within the criminal justice system (Rumney, 2006), and in society more broadly.

Although it is difficult to accurately determine the prevalence of false allegations of rape (Rumney, 2006), a 2013 Crown Prosecution Service study in the UK highlighted the small number of prosecutions for making false allegations of rape, especially when compared with prosecutions for rape (Levitt & Crown Prosecution Service Equality and Diversity Unit, 2013). However, it is suggested that a strategy involving the threat of a false allegation is one that is likely to have maximum impact when used by a woman because of existing legal and social definitions and understandings of sexual violence, i.e., men as perpetrators and women as victims. Therefore, while the same threats of a false rape allegation could be made by a man in respect of a woman, the woman concerned may not believe there would be real consequences for her as a result. For men, however, the potential for such a threat to become a reality may be particularly coercive because of the damaging consequences that could occur.

It is true that there are undoubtedly still issues around women who report sexual violence being believed (see, e.g., Bahadur, 2016; Jordan, 2004). However, a report of rape is (quite rightly) expected to at least involve a police investigation and, depending on the available evidence, potentially a criminal trial. There is also likely to be a substantial amount of emotional distress experienced by a man under investigation in the context of a false allegation due to the potential stigma and reputational ruin associated with being considered a “rapist” (Levitt & Crown Prosecution Service Equality and Diversity Unit, 2013; Wells, 2015). Societal perceptions around sexual violence perpetrators are only likely to enhance this further, with recognition of men as perpetrators and women as victims much more common than any other victim–perpetrator paradigm (Weare, 2018). This is understandable, with evidence consistently highlighting women as disproportionately experiencing sexual violence from men. Nevertheless, when taking all of this into account it is clear why women threatening false allegations of rape is a “gendered” coercive strategy, as well as being one that may be particularly powerful. While this strategy was only reported by two men, the complex nature of cases involving threats of/false rape allegations (Levitt & Crown Prosecution Service Equality and Diversity Unit, 2013) means that this is an issue that would benefit from further research in the context of it as a strategy used by sexually aggressive women. In exploring this issue further, it should not, however, be used to dismiss or undermine the experiences of women who experience sexual violence.

#### Exploitation of Women’s Roles as Mothers

More frequently, men reported women exploiting their roles as mothers or mothers-to-be, for example by threatening to negatively interfere in the men’s relationships with their children, harming the fetus while pregnant, or terminating the pregnancy. Seven men reported this strategy being used against them; for example: “[s]aid that she would stop all access to see my children” and “said she’d get an abortion if I didn’t have sex with her.”

As an institution, motherhood has been argued to be patriarchal and oppressive (Rich, 1995), requiring women to meet stereotypes around “good” mothering, and viewing as deviant those who do not (see, e.g., Roberts, 1993). Women’s role as mothers has also been documented as being used against them in the context of domestic abuse and coercive control perpetrated by men (Weissman, 2009). However, the individual experiences of women as mothers are not homogenous and include instances where women use their role as mothers, and primary caregivers, to “manage” their children and act as gatekeepers or influencers in the father–child relationship (see, e.g., Allen & Hawkins, 1999). In the context of the findings presented here, there is evidence that some women use their roles as mothers as a coercive strategy in relation to compelled penetration. In doing so, it appears that they are creating and exploiting a power hierarchy where they use their gendered role as mothers to solidify control over men’s behavior and coerce them into intercourse. While this specific strategy was reported relatively infrequently, the reoccurring and similar nature of the men’s experiences makes future consideration of this “gendered” strategy necessary.

### Conclusions

Based on quantitative and qualitative data provided by men who have experienced compelled penetration, the study reported on in this article evidences for the first time in the UK the experiences of men who have been FTP a woman. In doing so, the study demonstrates the range and frequency of aggressive strategies used by women, finding that women most frequently use coercive strategies, take advantage of men’s intoxication, and use force and threats of physical harm. Most significantly, for the first time, the findings highlight that some women use multiple aggressive strategies within one incident of compelled penetration, and that some women use particularly “gendered” strategies of threatening to make false rape accusations and exploiting their roles as mothers to threaten negative interference in the father–child relationship.

While being both novel and significant as the first study in the UK to specifically explore FTP cases, this research does have limitations. The participants self-reported their experiences, and thus there is a risk of reporting bias. Indeed, it was not possible, for example, to ascertain whether there had been bidirectional violence. In addition, the self-selecting nature of participants meant that the participant group was not representative, and, for example, ethnicity, religion, and socioeconomic background were not considered. Thus, future research would benefit from considering issues around intersectionality. Furthermore, due to the method of data collection, i.e., an online survey, the issues of data subjectivity, reliability and validity could be raised, with the possibility that some participants did not in fact experience compelled penetration, but instead completed the survey for “entertainment purposes.” This limitation, while perhaps more likely to occur in the context of an online survey, is not confined to this method of data collection and can plague any method, including face-to-face interviews. The justifications for using this data collection method (noted earlier) overrode this particular limitation and where it was clear that participants were “hoaxers” these surveys were removed. Despite these limitations, the findings presented here can usefully be considered by practitioners within the criminal justice system in relation to developing education, understanding, and responses to this under-reported form of sexual violence.

It is clear that future research is necessary in relation to FTP cases to maximize understanding and to develop a larger evidence base in this area. More research on the aggressive strategies of female perpetrators, especially in relation to the novel “gendered” strategies identified here, would be helpful to develop better understanding around their use. It would be helpful for future studies to explore predictors of use in relation to the aggressive strategies discussed in this article. Potentially interesting predictors could relate to female perpetrators’ experiences of non-consensual sexual activity themselves, their attitudes about gender roles, and their cultural, religious, and socioeconomic backgrounds. Interviews with male victims and with female aggressors would also allow fuller understanding of the complexities of this form of sexual violence.

As noted at the beginning of the article, FTP cases cannot be prosecuted under the offense of rape within the UK, instead being prosecuted under other “less serious” offenses. Justification for this approach has been based on the premise that compelled penetration is less harmful or detrimental to men than rape (see, e.g., Cowan, 2010; Home Office, 2000; Weare, 2018). Therefore, future research on the consequences of compelled penetration for men who experience it would be helpful in considering the need for legal reform. Similarly, consideration of the legal implications of, and challenges posed by, FTP cases, while outside of the scope of this article, could helpfully form the basis of the future research agenda in this area. Finally, future studies involving representative sampling would be useful for determining valid prevalence rates in the UK for this form of sexual violence.

This, and any future research around FTP cases, should not be viewed “as an attempt to upend a women’s rights agenda focused on male-perpetrated sexual victimization. [Nor should it] negate concern about other forms of abuse” (Stemple et al., 2016, p. 2). Indeed, it is clear that women are disproportionately affected by sexual violence perpetrated by men. However, this study highlights a need for women’s sexual aggression to be incorporated into the mainstream of research on sexual violence, as well as feminist criminological and legal research. In doing so, gender as a variable in cases of sexual violence should not be ignored, not least because “sexual aggression is not gender-neutral in its prevalence…or in its meanings and consequences” (Muehlenhard, 1998, p. 43). Rather, “feminist imperatives to undertake intersectional analyses, to take into account power relations, and to question gender-based stereotypes” (Stemple et al., 2016, p. 2) are needed, as well as analyses that go “beyond gender alone and look at other variables that may interact with gender” (Muehlenhard, 1998, p. 43). This will allow a multifaceted analysis of FTP cases as a specific form of sexual violence to be undertaken, in a way that does not undermine the experiences of women as victims of sexual violence.

## Footnotes

1 There are different legal definitions of rape in Scotland and Northern Ireland, but both are still defined in a way that means only men can be recognized as principal offenders. References to law in the article will be to that within England and Wales.

2 NVivo is a software package that is purpose-built for qualitative and mixed-methods research. It provides a platform by which the data can be stored, organized, and analyzed—http://www.qsrinternational.com/nvivo/what-is-nvivo.
